# Effects of grip variation on biceps brachii and brachioradialis electromyographic activity during concentric and eccentric phases of the barbell curl exercise

**DOI:** 10.1186/s13102-026-01867-7

**Published:** 2026-07-10

**Authors:** Abdullah Uysal, Batuhan Karaduman, Recep Soslu, Sefa Lök, Karuppasamy Govindasamy, Josyula Tejaswi, Hüseyin Şahin Uysal

**Affiliations:** 1 Department of Coaching Education, Karamanoğlu Mehmetbey University, Faculty of Sports Sciences, Karaman, 70200 Turkey; 2 Department of Coaching Education, Konya Selçuk University, Faculty of Sports Sciences, Konya, Turkey; 3 Department of Sports, Recreation and Wellness, Symbiosis International (Deemed University), Hyderabad Campus, Modallaguda (V), Nandigama (M), Rangareddy, Hyderabad, Telangana 509217 India; 4 Symbiosis School of Sports Sciences, Symbiosis International (Deemed University), Gram: Lavale, Tal: Mulshi, Pune, Maharashtra 412115 India; 5 Burdur Mehmet Akif Ersoy University, Faculty of Sport Sciences, Burdur, 15030 Turkey

**Keywords:** Resistance exercise, Surface electromyography, Barbell biceps curl, Biceps brachii, Brachioradialis, Bayesian analysis

## Abstract

This study investigated the effects of grip variations (supinated, semi-supinated, and neutral) and contraction phases (concentric, eccentric, and combined) on surface electromyographic (sEMG) activity of the biceps brachii (BB) and brachioradialis (BRR) during the barbell biceps curl (BBC). Twelve resistance-trained males performed BBCs under three grip conditions. sEMG activity was recorded bilaterally following SENIAM guidelines and normalized to maximal voluntary isometric contractions. A Bayesian framework evaluated activation differences within and between muscles across contraction phases. Concentric contractions elicited greater activation than eccentric phases in both muscles, while combined concentric–eccentric (CON/ECC) repetitions produced the highest activation, with strong to extreme evidence for hypothesis H1. Between-muscle comparisons showed higher BRR activation than BB during the eccentric phase, whereas concentric and CON/ECC actions yielded similar activations, supporting anecdotal evidence. These findings indicate that contraction phase and grip–bar configuration influence acute neuromuscular activation patterns during the barbell biceps curl. Across the examined conditions, combined concentric–eccentric repetitions elicited the greatest normalized activation in both muscles, whereas brachioradialis activation was greater than biceps brachii activation during eccentric actions, particularly in the supinated and semi-supinated grip–bar configurations. These results characterize acute muscle-activation patterns during the tested barbell curl conditions.

## Introduction

Resistance training is one of the most effective exercise modalities for enhancing athletic performance and overall health across various populations [1]. The benefits are well-documented, especially in increasing muscle strength, promoting hypertrophy, and improving neuromuscular function [2, 3]. These adaptations depend not only on training volume and loading parameters but also on the biomechanical characteristics of the exercise, its execution technique, and joint positioning [4]. In addition, exercise-induced muscle activation patterns are highly task-specific, meaning that even subtle variations in grip or joint angle may lead to distinct neural adaptations [5]. However, acute surface electromyographic (sEMG) activity should be interpreted as an indicator of neuromuscular activation during the tested task and does not, by itself, establish subsequent hypertrophic adaptation. Recent studies conducted across different resistance-exercise models further indicate that acute muscle-activation and performance responses may vary according to exercise configuration, loading conditions, and the specific characteristics of the task performed [6–8].

Understanding how muscle activation levels vary across different exercise variations is essential for characterizing the acute neuromuscular demands of resistance exercises [9–11]. Performing the same exercise with varying grip positions can substantially alter target muscle activation (ES = − 0.38 to 0.49) [12]. Considering the exercises performed for the upper extremity, previous studies have mainly focused on three primary forearm positions (supination, pronation, and neutral) while overlooking intermediate positions such as semi-supination [10]. However, even small changes in forearm rotation can modify the moment arm and mechanical advantage, thereby influencing activation in both the biceps brachii (BB) and brachioradialis (BRR) muscles [11]. This is particularly relevant for practitioners, since forearm rotation is frequently adjusted in resistance training to reduce discomfort, optimize mechanical leverage, or selectively target muscle groups [13]. In addition to forearm position, bar configuration may influence joint alignment and muscle activation patterns during resistance exercise [9, 13, 14]. In practical resistance-training settings, supinated curls are commonly performed with a straight bar, semi-supinated curls with an EZ bar, and neutral-grip curls with a hammer curl bar. Therefore, the present conditions were considered integrated grip–bar configurations rather than isolated manipulations of forearm rotation. This approach permits examination of commonly used exercise variations, although the independent effects of grip orientation and bar type cannot be separated within the present design. Among the various resistance training exercises, the barbell biceps curl provides a controlled and widely adopted movement pattern where such grip–bar configurations can be systematically examined [10, 11].

The barbell biceps curl (BBC) is an ideal model for investigating the activation of elbow flexors such as the BB and BRR [9, 11]. Nevertheless, the literature lacks comprehensive assessments that simultaneously examine the effects of different grip–bar configurations and movement phases (concentric [CON] and eccentric [ECC]) on electromyographic (EMG) activity. The ECC phase, characterized by higher force production capacity, lower energy cost, and distinct motor unit recruitment patterns, may produce markedly different activation profiles than the CON phase [15]. Eccentric contractions are also commonly included in resistance-training and rehabilitation programmes because they impose distinct mechanical and neuromuscular demands [15, 16]. However, the present study evaluates acute EMG responses and does not assess muscle remodeling, tendon adaptations, or rehabilitation outcomes. Previous EMG studies on the barbell biceps curl have shown phase- and grip-dependent differences in muscle activation. Marcolin et al. [11] found lower eccentric than concentric activation in both BB and BRR, while Coratella et al. [9] reported greater concentric activation with supinated grip and higher anterior deltoid activity in eccentric phases with pronated and neutral grips. However, previous protocols have primarily examined a limited range of forearm positions and have not fully addressed intermediate configurations such as semi-supination. In addition, because bar type and forearm position may be varied together in practical curl variations, the resulting findings should be interpreted as reflecting combined grip–bar configurations rather than isolated grip effects [9, 11, 13, 14]. Both highlight the need for more comprehensive protocols, including intermediate positions [9, 11].

On the other hand, most previous research has relied on frequentist statistical approaches, interpreting results through p-values [9, 11, 17]. The p-value approach merely indicates whether the null hypothesis can be rejected, without directly quantifying the strength of evidence [18]. In small samples, the risk of Type I and II errors is further exacerbated [19]. Bayesian statistics, by contrast, allow for direct probability comparisons between competing hypotheses and express the strength of evidence through Bayes factors (BF10), offering more transparent and more interpretable results. This approach has gained increasing traction in sports and exercise science, providing a more nuanced framework for evaluating physiological responses and training interventions [20].

This study aimed to compare sEMG activation levels of the BB and BRR muscles during BBC exercise across three forearm positions (supinated, semi-supinated, and neutral) and three contraction phases (CON, ECC, CON/ECC). The following statement constituted the problem statement for this study: How do different grip positions (supination, semi-supination, neutral) and contraction phases (concentric and eccentric) affect the activation levels of the biceps brachii and brachioradialis muscles in the barbell biceps curl exercise?

## Methods

### Study design

This study used a randomized, repeated-measures crossover laboratory design in which each participant completed all three grip–bar configurations. The Quality Output Checklist and Content Assessment (QuOCCA) was used to improve the quality of the study’s reporting. In addition, the Surface EMG for Non-invasive Assessment of Muscles (SENIAM) guidelines were followed for EMG sensors and sensor placement procedures [21]. Details of the study files are available openly through OSF (https://osf.io/6emxt/).

### Participants

The required sample size was determined using G*Power software (version 3.1.9.7, University of Düsseldorf, Düsseldorf, Germany). The previous study’s findings were used as a reference to establish the appropriate sample size for the current research [11]. A *priori* power analysis for repeated-measures ANOVA with three within-subject conditions (grip positions), a medium effect size (ES = 0.25), α = 0.05, power (1-β) = 0.80, and an assumed correlation of 0.5 among repeated measures indicated that a minimum of 12 participants was sufficient. All participants had been regularly engaging in resistance training for at least one year, with a minimum weekly frequency of 3 sessions. None of them were classified as professional or elite athletes, nor were they sedentary. Therefore, the sample represented non-elite, physically trained individuals with consistent strength training backgrounds. Accordingly, twelve male volunteers (mean age = 21.77 ± 1.42 years; height = 176.92 ± 5.68 cm; body mass = 80.77 ± 9.97 kg; Body mass index = 25.82 ± 3.03 kg/m²) were recruited. All participants had at least one year of resistance training experience and had regularly included BBC exercises in their routines for the preceding three months, training an average of 3–4 times per week. Participants were clinically healthy, non-smokers, and free from any known neurological, cardiovascular, or upper limb musculoskeletal disorders for the past year. Exclusion criteria included recent injury, use of performance-enhancing drugs, or engagement in intense exercise within 48 h before testing. The order of grip conditions was randomized using a computer-generated block randomization scheme (block size = 3) to mitigate learning effects and order bias. The study protocol was approved by the Non-Interventional Clinical Research Ethics Committee of Selçuk University, Faculty of Sport Sciences (Approval No: E-40990478-050.99-757512) and registered on the Open Science Framework (https://osf.io/6emxt/). All procedures complied with the Declaration of Helsinki, and written informed consent was obtained from each participant before study commencement.

### Experimental procedures

All participants attended two laboratory sessions, both conducted between 09:00 a.m. and 11:00 a.m. to minimize the effects of circadian fluctuations on performance. This time frame was chosen based on previous literature recommending morning hours for reliable assessments of muscle strength and motor performance, as these measures are less likely to be influenced by diurnal variation during this period [22]. In the first session, participants’ anthropometric data, including height and body weight, were measured using a stadiometer (SECA-Mod. 220, Seca GmbH & Co. KG., Hamburg, Germany), and body mass index was calculated using the standard kg/m² formula [23]. Subsequently, the one-repetition maximum (1RM) load for BBC exercises across three grip–bar configurations (supinated, semi-supinated, and neutral) was estimated using the Brzycki equation [24]:

$$1\mathrm{RM}\;=\;\mathrm{Lifted}\;\mathrm{weight}\;/\;\left[1.0278\;-\left(0.0278\;\times\;\mathrm{Number}\;\mathrm{of}\;\mathrm{repetitions}\right)\right]$$

To estimate 1RM, participants selected a suitable weight after a warm-up set. They performed repetitions with this weight until fatigue was reached. Participants’ weight and maximum repetitions were recorded and used to estimate 1RM. A 10-minute rest was provided between each grip–bar configuration. Each forearm position was paired with a bar configuration commonly used in resistance-training practice: a straight bar was used for the supinated condition, an EZ bar for the semi-supinated condition, and a hammer curl bar for the neutral condition. The deliberate pairing of bar type with each grip position was chosen to enhance ecological validity by allowing participants to perform each variation using commonly adopted and anatomically natural configurations in resistance-training practice, rather than isolating pure forearm rotation under artificial constraints. This approach was intended to examine practical grip–bar configurations and to accommodate the customary wrist and elbow alignment associated with each variation. Previous research has reported that bar type and joint position may influence muscle activation levels during resistance exercises [13, 14]. Accordingly, the bar–grip pairings were standardized in advance to ensure that all participants performed the same practical configuration for each condition. Hand placement was standardized according to the predefined grip locations of each bar configuration and was visually checked by the researchers before and during each set. Participants were instructed to perform the movement through a consistent and comfortable full range of motion while avoiding compensatory trunk or shoulder movements. Importantly, the protocol was not designed to isolate the independent effect of forearm position from that of bar type. Therefore, the findings should be interpreted as reflecting combined grip–bar configurations, and any observed differences in sEMG activity cannot be attributed exclusively to grip orientation or bar design.

Accordingly, the present design prioritized the examination of practical and ecologically valid curl variations rather than the isolated biomechanical effect of forearm rotation. The independent contributions of forearm position and bar geometry should be examined in future studies using factorial designs in which these variables are manipulated separately. At the end of the session, participants were familiarized with the testing procedures planned for the second session, including movement technique, range of motion, postural alignment, and repetition tempo using a metronome set at 20 beats per minute (BPM). During the second session, participants performed a standardized 10-minute warm-up protocol, which consisted of 5 min of cycling on a bicycle ergometer at 50 watts, followed by dynamic upper limb stretches (e.g., shoulder rotations, arm circles, wrist mobilizations). This was followed by a specific warm-up involving 2 sets of 6 repetitions of the BBC at approximately 40–50% of their 1RM.

To collect EMG signals, Ag/AgCl pre-gelled bipolar surface electrodes (20 mm inter-electrode distance) were placed on the dominant arm over the BB and BRR muscles according to the SENIAM guidelines [25]. The skin was shaved and cleaned with an alcohol-based solution to reduce impedance. EMG data were recorded at a sampling frequency of 4000 Hz using a 16-channel wireless EMG system (Noraxon Inc., Scottsdale, AZ, USA) and transmitted in real time to a laptop computer (HP Probook 450 G6, Intel Core i7, Palo Alto, California, USA). Simultaneously, joint kinematics and movement phases (CON/ECC) were recorded using a 16-sensor wireless MyoMotion motion analysis system (Noraxon Inc., Scottsdale, AZ, USA). Sensors were attached to the dominant arm, specifically to the midpoint between the shoulder and elbow (humerus) and the distal posterior region of the forearm (ulna), following the manufacturer’s instructions (Appendix C). System calibration was performed using the anatomic position and upright standing reference positions, with 0° of elbow flexion set as baseline. The calibration was considered valid when the deviation remained within a ± 2° tolerance. Kinematic data were sampled at 200 Hz and synchronized with EMG recordings using the MyoSync device (Noraxon Inc., USA).

To normalize EMG signals, three MVICs were performed for both BB and BRR muscles. Each MVIC trial was separated by a 5-minute rest interval. Participants sat upright with their dominant arm fixed at 90° elbow flexion and the forearm in a neutral position. Manual resistance was applied to the distal third of the forearm by the investigator while participants exerted maximum effort in static elbow flexion for 5 s [26–28]. An external dynamometer or load cell was not used; therefore, the manual-resistance MVIC procedure may have introduced tester-dependent variability and limited the robustness of between-trial standardization. Manual resistance applied by an experienced examiner has nevertheless been used for MVIC normalization in EMG research [27]. To reduce potential variability, all MVIC trials were conducted by the same experienced examiner throughout data collection, and the trial eliciting the highest EMG activity was retained as the reference value for normalization. Nevertheless, the absence of an external dynamometer or load cell should be considered a limitation of the present study. Raw EMG data were processed using a band-pass filter (20–500 Hz), full-wave rectified, and smoothed using an RMS algorithm with a 100-ms moving window. The EMG signals for BB and BRR were then normalized to the average peak RMS value obtained from a 500-ms segment of the MVIC. Artifacts in EMG signals were identified based on visual inspection of the raw signal and the presence of marked anomalies such as sudden peak values ( > ± 2.5 SD), low signal amplitude (< 0.02 mV), or discontinuities in the signal. Suspicious signals were re-examined by the researcher after an automatic artifact filter was applied, and any repetitions found to be erroneous were repeated in accordance with the study protocol.

**Fig. 1:**
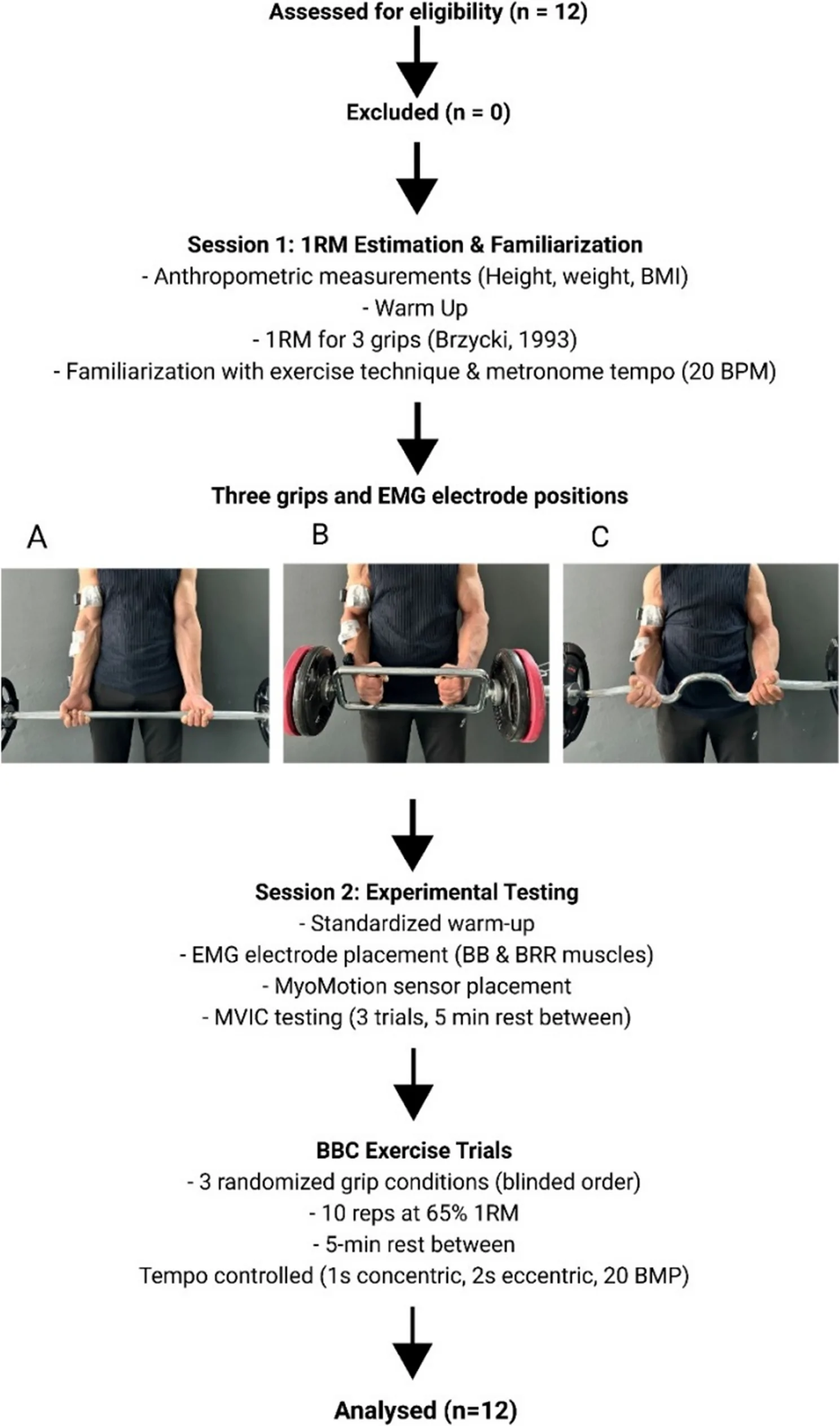
Experimental design of the study. *Legend.* **A**: Supinated grip; **B**: Neutral grip; **C**: Semi-supinated grip; BBC: Barbell biceps curl; BB: Biceps brachii; BR: Brachioradialis; BMI: Body mass index; EMG: Electromyography; MVIC: Maximum Voluntary Isometric Contraction; BMP: BPM: Beats per minute

Following normalization, participants performed the BBC exercise under each of the three grip–bar configurations in a randomized order determined by computer-generated block randomization. Participants could perceive the different bar types and hand positions; therefore, they were not considered blinded to the experimental conditions. However, participants were not informed of the specific study hypotheses or expected muscle-activation differences across conditions before testing. Each exercise set consisted of 10 repetitions at 65% of the participant’s 1RM. Prior to testing, a pre-assessment confirmed that all participants were capable of performing 10 repetitions at this load with proper technique. Sets containing technical errors were repeated, and only valid repetitions were included in the analysis. A 5-minute passive rest was provided between each set. All exercise repetitions were performed with the aid of a metronome set to a tempo of 20 BPM. This tempo allowed each repetition to be completed in approximately 3 s (1 s CON and 2 s ECC). This rhythm provided standardization of movement tempo and ensured suitability for time-based analyses. To prevent compensatory movements, trunk sway, knee flexion, and overall posture were visually monitored by the researchers throughout the exercises. The data collection process was carried out by two researchers (A.U. and B.K.) who were < u> not involved in the subsequent statistical analyses. The researchers who performed the statistical analysis (R.S. and H.Ş.U.) were not involved in data collection (Fig. 1).

### Data processing procedures

Raw EMG signals were collected at a sampling frequency of 4000 Hz using a wireless surface EMG system (Noraxon Inc., Scottsdale, AZ, USA). Kinematic data were simultaneously recorded at 200 Hz using the MyoMotion motion analysis system. The datasets were synchronized in real time via Bluetooth using the MyoSync interface and transferred to a laptop computer (HP Probook 450 G6, Intel Core i7). All data were recorded and processed using MyoResearch 3.16.32 software (Noraxon Inc., Scottsdale, AZ, USA). EMG signals were normalized to the MVIC values recorded for each participant. The reference value for normalization was defined as the average peak RMS value within a 500 ms window during the MVIC trial with the highest activation. Signal processing included a band-pass filter (20–500 Hz), full-wave rectification, and RMS smoothing using a 100 ms moving window. MVIC trials exhibiting artifacts were excluded and repeated to ensure data quality [29].

### Data analysis

Statistical analyses were performed using Bayesian paired-samples t-tests and Bayesian repeated-measures one-way ANOVA to examine sEMG activity during the BBC exercises across the tested grip–bar configurations and contraction phases. Bayesian paired-samples t-tests were used for between-muscle comparisons of the BB and BRR within each grip–bar configuration and contraction phase, because both measurements were obtained from the same participants under the same experimental condition. Bayesian repeated-measures one-way ANOVA was used to examine differences across contraction phases (CON, ECC, and CON/ECC) within each muscle and grip–bar configuration. The normalized RMS amplitude values obtained from the BB and BRR muscles were designated as the dependent variables. Before statistical analysis, all EMG data were normalized to each participant’s peak MVIC to control for inter-individual variability. Analyses were performed using JASP statistical software (version 0.19.3.0, Amsterdam, The Netherlands), employing default Cauchy priors centered at zero (*r* = 0.5) for the paired-samples t-tests. The results were interpreted using Bayes factors (BF₁₀), with the strength of evidence supporting the alternative hypothesis (H₁) or the null hypothesis (H₀) classified according to established interpretative thresholds [30]: extreme (BF₁₀ > 100), very strong (30 ≤ BF₁₀ < 100), strong (10 ≤ BF₁₀ < 30), moderate (3 ≤ BF₁₀ < 10), and anecdotal (1 ≤ BF₁₀ < 3) for H₁. Conversely, support for H₀ was interpreted as extreme (BF₁₀ < 0.001), very strong (0.001 ≤ BF₁₀ < 0.03), strong (0.03 ≤ BF₁₀ < 0.1), moderate (0.1 ≤ BF₁₀ < 0.3), and anecdotal (0.3 ≤ BF₁₀ < 1).

## Results

### Adherence to intervention

Twelve male participants with resistance training experience were included in this study. Participants demonstrated 100% adherence to the intervention. No adverse events or injuries were observed during the intervention. Additionally, no software-related issues were encountered.

### Results for between-muscle comparisons

#### Results of muscle activation in concentric and eccentric phases

**Fig. 2:**
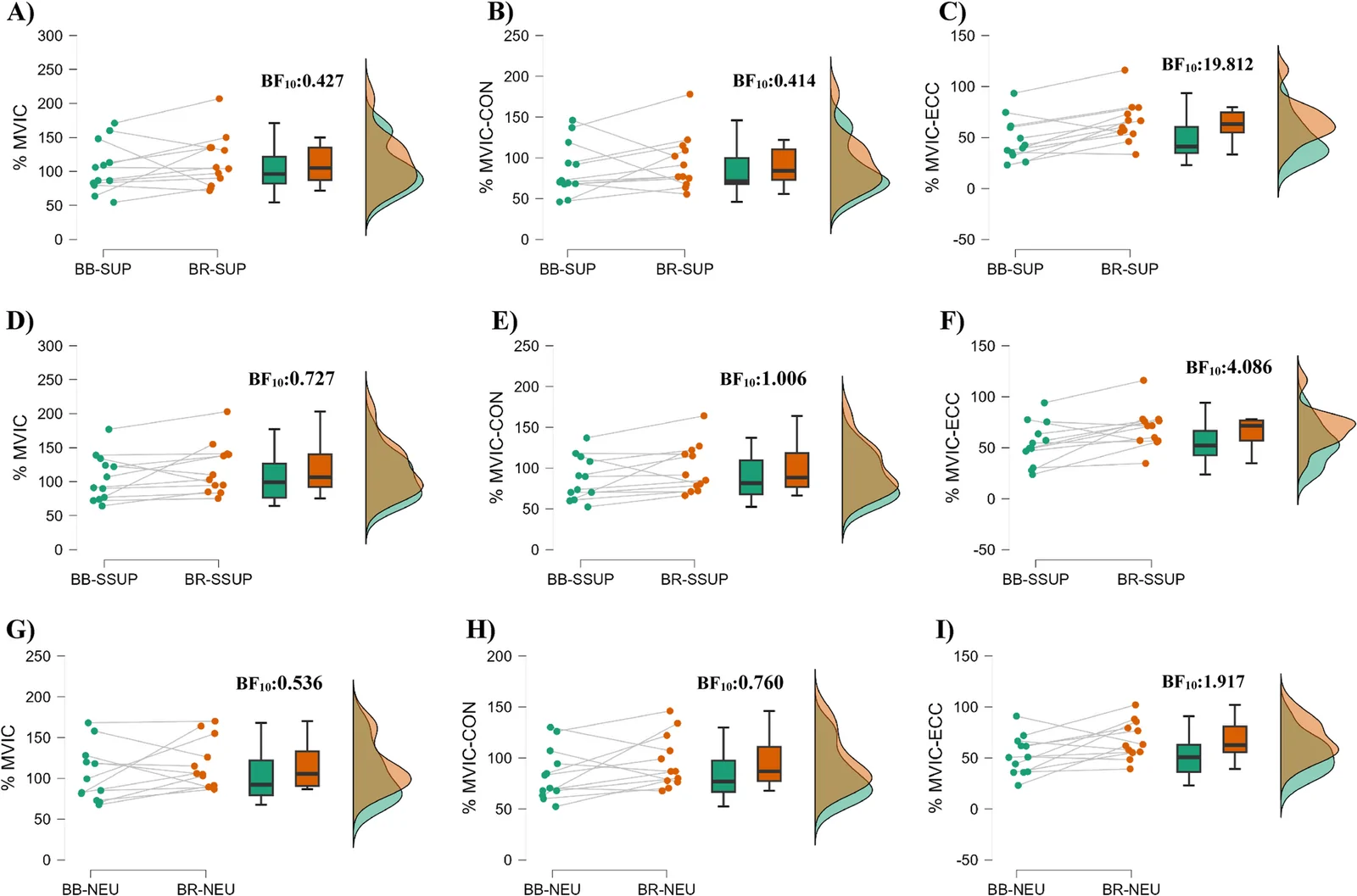
Comparison of muscle activation in biceps curl variations. *Legend.* BB: Biceps brachii; BR: Brachioradialis; SUP: Supinated; SSUP: Semi-supinated; NEU: Neutral; MVIC: Maximum voluntary isometric contraction; CON: Concentric; ECC: Eccentric; BF10: Bayes Factor. **A**: Comparison of muscles in supinated position in the concentric and eccentric phases; **B**: Comparison of muscles in supinated position in the concentric phase; **C**: Comparison of muscles in supinated position in the eccentric phase; **D**: Comparison of muscles in semi- supinated position in the concentric and eccentric phases; **E**: Comparison of muscles in semi- supinated position in the concentric phase; **F**: Comparison of muscles in semi- supinated position in the eccentric phase; **G**: Comparison of muscles in neutral position in the concentric and eccentric phases; **H**: Comparison of muscles in neutral position in the concentric phase; **I**: Comparison of muscles in neutral position in the eccentric phase

Comparing the combined mean of the BB and BRR muscles across the CON and ECC phases revealed anecdotal evidence supporting hypothesis H0 in supination (BF10 = 0.427), semi-supination (BF10 = 0.727), and neutral (BF10 = 0.536) grips. Although BRR means were higher than BB means across all three grip positions, no significant differences were observed between groups. Details regarding muscle activation during the CON and ECC phases are presented in Table 1; Fig. 2.

**Table 1:** Bayesian paired-samples comparisons of biceps brachii and brachioradialis activation across contraction phases and grip–bar configurations

Phase	Group 1				Group 2				Bayesian T-Test		
	Variable	N	Mean	SD	Variable	N	Mean	SD	BF₁₀	Hypothesis	Level of evidence
CON/ECC	BB-SUP	12	105.05	37.39	BR-SUP	12	115.08	38.91	0.427	H_0_	Anecdotal
	BB-SSUP	12	105.95	34.02	BR-SSUP	12	118.61	37.50	0.727	H_0_	Anecdotal
	BB-NEU	12	104.30	34.09	BR-NEU	12	116.65	30.39	0.536	H_0_	Anecdotal
CON	BB-SUP	12	59.63	20.56	BR-SUP	12	67.42	19.25	0.414	H_0_	Anecdotal
	BB-SSUP	12	63.13	19.24	BR-SSUP	12	71.49	17.03	1.006	H_1_	Anecdotal
	BB-NEU	12	60.32	16.62	BR-NEU	12	70.76	18.39	0.760	H_0_	Anecdotal
ECC	BB-SUP	12	27.92	11.19	BR-SUP	12	37.97	10.05	19.812	H_1_	Strong
	BB-SSUP	12	31.17	10.22	BR-SSUP	12	42.37	10.04	4.086	H_1_	Moderate
	BB-NEU	12	29.56	8.077	BR-NEU	12	40.85	9.887	1.917	H_1_	Anecdotal

*Legend.* *BB* Biceps Brachii, *BR* Brachioradialis, *SUP* Supinated grip, *SSUP* Semi-supinated grip, *NEU* Neutral grip, *BF*₁₀ Bayes Factor (evidence in favor of H₁ relative to H₀), *SD* Standardd deviation, *N* Sample size

#### Results of muscle activation in concentric phases

Comparative analyses of the BB and BRR muscles during the CON phase revealed higher mean activation levels in favor of the BRR across all grip positions. However, Bayesian analysis results provided only anecdotal evidence for differences between groups. Anecdotal evidence favored hypothesis H_0_ in the supinated (BF_10_ = 0.414) and neutral (BF_10_ = 0.760) grips, while anecdotal evidence favored hypothesis H1 in the semi-supinated (BF_10_ = 1.006) grip. Details regarding muscle activation during the CON phase are presented in Table 1; Fig. 2.

#### Results of muscle activation in eccentric phases

Comparative analyses of the BB and BRR muscles during the ECC phase revealed higher mean activation values in favor of the BRR in all grip positions. However, Bayesian analysis findings provided varying levels of evidence across the different grips. In the supination grip, the BRR muscle showed higher activation than the BB, which was supported by strong evidence in favor of H_1_ (BF_10_ = 19.812). Similarly, the BRR showed higher activation in the semi-supination grip than the BB. These results provided moderate evidence in favor of H_1_ (BF_10_ = 4.086). On the other hand, the BRR showed higher mean activation in the neutral grip compared to the BB. However, this difference was only supported by anecdotal evidence in favor of H_1_ (BF_10_ = 1.917). Details regarding muscle activation during the CON phase are presented in Table 1; Fig. 2.

### Results for intragroup comparisons

#### Results on biceps brachii muscle activation

Bayesian One-Way ANOVA results revealed extreme evidence in favor of hypothesis H1 across contraction phases in all three grip positions (BF_incl_ = 1.36 × 10⁵ to 9.34 × 10⁵). Post-hoc analyses indicated muscle activation in the CON phase was higher than in the ECC phase for all three grip positions. Furthermore, CON/ECC revealed higher activation levels than CON and ECC phases. These results were supported by strong to extreme evidence in favor of hypothesis H_1_ across supination, semi-supination, and neutral grips (BF_incl_ = 24.863 to 9.34 × 10⁵). Details of the within-group comparisons for BB activation are presented in Table 2.

**Table 2:** Bayesian repeated-measures comparisons of contraction phases within each muscle across grip–bar configurations

Muscle	Bayesian Repeated-Measures One-Way ANOVA				*Bayesian paired-samples post-hoc comparisons*										
	Grip–bar configuration	Muscle contraction effect (BF_incl_)	Hypothesis	Level of evidence	Group1	N	Mean	SD	Group2	N	Mean	SD	BF_10_	Hypothesis	Level of evidence
Biceps Brachii	Supinated grip	136071.506	H_1_	Extreme	CON	12	59.633	20.566	ECC	12	27.925	11.194	185.291	H_1_	Extreme
					CON	12	59.633	20.566	CON/ECC	12	105.058	37.397	24.863	H_1_	Strong
					ECC	12	27.925	11.194	CON/ECC	12	105.058	37.397	1.53 × 10⁷	H_1_	Extreme
	Semi-supinated grip	397893.657	H_1_	Extreme	CON	12	63.133	19.249	ECC	12	31.175	10.224	412.634	H_1_	Extreme
					CON	12	63.133	19.249	CON/ECC	12	105.950	34.022	30.600	H_1_	Very strong
					ECC	12	31.175	10.224	CON/ECC	12	105.950	34.022	3.71 × 10⁷	H_1_	Extreme
	Neutral grip	934287.101	H_1_	Extreme	CON	12	60.325	16.629	ECC	12	29.567	8.077	1.699.662	H_1_	Extreme
					CON	12	60.325	16.629	CON/ECC	12	104.308	34.092	47.372	H_1_	Very strong
					ECC	12	29.567	8.077	CON/ECC	12	104.308	34.092	4.50 × 10⁷	H_1_	Extreme
Brachioradialis	Supinated grip	120679.886	H_1_	Extreme	CON	12	67.425	19.251	ECC	12	37.975	10.059	187.541	H_1_	Extreme
					CON	12	67.425	19.251	CON/ECC	12	115.083	38.913	31.102	H_1_	Very strong
					ECC	12	37.975	10.059	CON/ECC	12	115.083	38.913	1.03 × 10⁷	H_1_	Extreme
	Semi-supinated grip	253020.482	H_1_	Extreme	CON	12	71.492	17.036	ECC	12	42.375	10.045	430.649	H_1_	Extreme
					CON	12	71.492	17.036	CON/ECC	12	118.617	37.502	42.609	H_1_	Very strong
					ECC	12	42.375	10.045	CON/ECC	12	118.617	37.502	14.101.250	H_1_	Extreme
	Neutral grip	3.805 × 10^+ 6^	H_1_	Extreme	CON	12	70.767	18.396	ECC	12	40.850	9.887	323.884	H_1_	Extreme
					CON	12	70.767	18.396	CON/ECC	12	116.658	30.390	119.133	H_1_	Extreme
					ECC	12	40.850	9.887	CON/ECC	12	116.658	30.390	218.617.636	H_1_	Extreme

*Legend.* *BB* Biceps Brachii, *BR* Brachioradialis, *SUP* Supinated grip, *SSUP* Semi-supinated grip, *NEU* Neutral grip, *BF*₁₀ Bayes Factor (evidence in favor of H₁ relative to H₀), *SD* Standard deviation, *N* Sample size

#### Results on brachioradiles muscle activation

The Bayesian One-Way ANOVA results for BRR revealed extreme evidence in favor of hypothesis H1 across CON phases in all three grip positions (BF_incl_ = 1.21 × 10⁸ to 3.81 × 10⁶). Post-hoc analyses showed that muscle activation in the CON phase was higher than in the ECC phase for all three grip positions. Furthermore, CON/ECC elicited higher activation levels than the CON and ECC phases. These results were supported by evidence ranging from very strong to extreme in favor of hypothesis H1 for supination, semi-supination, and neutral grips (BF_10_ = 31.102 to 2.19 × 10⁸). Details of the within-group comparisons for BRR activation are presented in Table 2.

## Discussion

This study aimed to examine the effects of different grip positions (supinated, semi-supinated, and neutral) and contraction phases (CON, ECC, and CON/ECC) on sEMG activation of the BB and BRR. In response to the research question, our findings indicated that contraction phases play a decisive role in muscle activation. Specifically, CON contractions produced greater activation than ECC contractions, while CON/ECC repetitions yielded the highest activation for both muscles. Intermuscular comparisons revealed that the BRR exhibited greater activation than the BB during the ECC phase, while differences between the CON and CON/ECC muscles were only anecdotal. These results suggest that both grip positions and contraction types significantly influence muscle activation, with the BRR playing a dominant role, particularly during the ECC phase.

One of the essential findings of this study was that BRR produced greater muscle activation in the ECC phase compared to BB. The finding that the brachioradialis exhibits greater muscle activation during the eccentric phase than the biceps brachii may be attributable to various physiological and biomechanical factors underlying muscle function and contraction strategies. Research demonstrates that during eccentric contractions, muscles can generate significant amounts of force during lengthening, resulting in increased muscle tension and improved force production efficiency [31]. This is particularly noteworthy for the brachioradialis, which utilizes a unique anatomical position during eccentric tasks, providing an advantage over the biceps brachii. In addition to playing a key role in stabilizing the forearm during flexion movements, the brachioradialis also serves as a primary elbow flexor [32]. As the elbow approaches full extension during eccentric loading, the brachioradialis may achieve greater muscle activation due to its optimal positioning to resist the forces acting on the forearm [33]. In contrast, the biceps brachii may rely on a less optimal mechanical position during eccentric actions, resulting in relatively lower muscle activation as observed with surface electromyography (sEMG) [11]. Researchers have reported lower EMG activity of the biceps brachii compared to the brachioradialis during eccentric curls [11], which supports our findings. However, Coratella et al. [9] demonstrated that forearm position, by highlighting grip-related variations, may modulate the relative contribution of the elbow flexors. These results suggest that the dominance of the brachioradialis during eccentric curls is not merely a mechanical consequence of forearm rotation but also a neuromuscular strategy to optimize stability and efficiency during extension contractions.

The only anecdotal differences in activation between the BB and BRR in the CON and CON/ECC phases may be due to the synergistic work of the muscles, as well as the similar activation profiles of the grip and exercise variants. Marcolin et al. [11] reported that significant differences for the BB and BRR were observed only when compared to the upright position in the ez-bar and straight-bar variations, while activation was very similar between the ez-bar and straight-bar variations. This suggests synergy and neural sharing prevent muscle differences in these phases. Furthermore, a recent study demonstrated that hand position and elbow angle modulate the activation of these muscles, but this effect is particularly pronounced only at specific load and angle combinations [10].

Another important finding of the study relates to the within-muscle comparisons. Across the tested grip–bar configurations, normalized sEMG activity was greater during the CON phase than during the ECC phase in both the BB and BRR. This finding indicates that concentric and eccentric actions were associated with different acute activation patterns under the present experimental conditions. Previous literature has shown that eccentric contractions differ from concentric contractions in terms of force-generating capacity, energetic cost, and motor-unit behavior [15, 34–38]. For example, a recent meta-analysis reported that maximal eccentric force may be substantially greater than maximal concentric force, which may influence the mechanical conditions present during transitions between contraction phases [34]. In addition, eccentric actions have been associated with distinct muscle–tendon behavior and lower fatigue responses compared with concentric actions [35, 37, 38]. However, elastic energy utilization, muscle-fiber recruitment, neural drive, force output, and fatigue were not directly assessed in the present study. Therefore, these mechanisms should be considered possible physiological interpretations rather than direct explanations of the observed sEMG differences. The greater normalized activation observed during the CON phase may be consistent with differences in contraction-specific neuromuscular demands, but direct confirmation would require concurrent assessment of force production, muscle–tendon behavior, motor-unit activity, and fatigue-related variables in future studies.

Finally, the highest muscle activation in our study with combined (CON/ECC) repetitions can be explained by the longer repetition duration compared to the other phases (CON and ECC), thus increasing the total muscle activation time. The sequential occurrence of the CON and ECC phases allows motor units to remain engaged for more extended periods, thus increasing the duration and intensity of neuromuscular stimulation. The literature indicates that changes in muscle length and the sequential application of different contraction types create a strong stimulus for hypertrophy by increasing mechanical tension and metabolic stress in the muscle-tendon unit [5, 16]. Existing mechanisms may provide a rationale for the highest muscle activation results in the CON/ECC phase.

Several limitations should be acknowledged. First, the exclusive use of surface electromyography limited the assessment of deep muscles such as the brachialis, which may play a compensatory role during elbow flexion. Second, the inclusion of only healthy, resistance-trained young males restricts the generalizability of the findings to other populations. Third, inter-individual differences related to electrode placement, subcutaneous tissue thickness, and biomechanics may have contributed to variability in EMG measurements. In addition, grip width and handle thickness were not controlled, although these factors may influence muscle activation patterns. Because grip orientation and bar type were varied simultaneously, the independent contribution of each factor to the observed activation patterns could not be determined. This issue is relevant because previous studies suggest that both forearm position and exercise equipment may influence muscle activation during resistance exercise [9–11, 13, 14]. Although EMG normalization was performed according to SENIAM recommendations, the absence of an external dynamometer for MVIC assessment may have introduced minor inter-trial variability. Future studies should include diverse populations, alternative bar configurations, and advanced techniques such as intramuscular EMG or ultrasound-based methods to further clarify intermuscular activation patterns. Therefore, the present findings should be interpreted as reflecting grip–bar configurations commonly used in applied resistance training settings rather than the isolated effects of forearm rotation alone.

## Conclusion

The present study demonstrated that grip variation and contraction phase exert distinct influences on the activation of the BB and BRR during the barbell biceps curl. Bayesian comparisons revealed that concentric contractions consistently elicited higher muscle activation than eccentric contractions. In contrast, combined concentric–eccentric movements produced the most significant overall activation levels with extreme evidence across all grip positions. In intermuscular comparisons, BRR generally showed higher mean activation than BB, particularly during the eccentric phase, where strong to anecdotal evidence supported its dominance in supinated and semi-supinated grips. However, concentric activation patterns indicated only anecdotal differences between the muscles across grips. These findings suggest that BB and BRR are effectively recruited during supinated, semi-supinated, and neutral grips; their relative contributions vary according to contraction phase. BRR plays a more prominent role in eccentric actions. Overall, the present findings characterize acute biceps brachii and brachioradialis activation patterns across the tested grip–bar configurations and contraction phases during the barbell biceps curl.

## Practical application

The present findings describe acute normalized sEMG activation patterns of the biceps brachii and brachioradialis during barbell curl exercise performed under different grip–bar configurations and contraction phases. Across the examined conditions, combined concentric–eccentric repetitions elicited the greatest normalized activation in both muscles. In addition, brachioradialis activation was greater than biceps brachii activation during eccentric actions, particularly in the supinated and semi-supinated grip–bar configurations. These findings may assist practitioners in identifying the acute activation characteristics of the tested barbell curl variations. However, the present results should not be interpreted as evidence of superior hypertrophy, reduced injury risk, tendon adaptation, improved muscle recovery, prevention of muscle imbalances, or rehabilitation effectiveness, as these outcomes were not directly assessed.

## Data Availability

The datasets generated and/or analyzed during the current study are available in the OSF repository ( https://osf.io/6emxt/ ).

## Abbreviations

EMG: Electromyographic
sEMG: Surface electromyographic
BB: Biceps brachii
BRR: Brachioradialis
BBC: Barbell biceps curl
MVIC: Maximal voluntary isometric contraction
RMS: Root mean square
1RM: One-repetition maximum
BPM: Beats per minute
BF_10_: Bayes Factors
CON: Concentric
ECC: Eccentric
SENIAM: Surface EMG for non-invasive assessment of muscles Guidelines
QuOCCA: The Quality Output Checklist and Content Assessment
OSF: Open Science Framework

## Code availability

Code lines can be obtained by contacting the corresponding researcher.
